# Impact of Sustained Exogenous Irisin Myokine Administration on Muscle and Myocyte Integrity in Sprague Dawley Rats

**DOI:** 10.3390/metabo12100939

**Published:** 2022-10-03

**Authors:** Foad Alzoughool, Mohammad Borhan Al-Zghoul, Bayan Y. Ghanim, Manar Atoum, Yousef Aljawarneh, Nasir Idkaidek, Nidal A. Qinna

**Affiliations:** 1 Department of Medical Laboratory Sciences, Faculty of Applied Medical Sciences, The Hashemite University, Zarqa 13133, Jordan; 2 Division of Health Sciences, Fujairah Women’s College, Higher Colleges of Technology, Fujairah 1626, United Arab Emirates; 3 Basic Veterinary Sciences, School of Veterinary Medicine, Jordan University of Science and Technology, Irbid 22110, Jordan; 4 University of Petra Pharmaceutical Center (UPPC), University of Petra, Amman 11196, Jordan; 5 Department of Pharmaceutics and Pharmaceutical Technology, Faculty of Pharmacy and Medical Sciences, University of Petra, Amman 11196, Jordan; 6 Department of Pharmacology and Biomedical Sciences, Faculty of Pharmacy and Medical Sciences, University of Petra, Amman 11196, Jordan

**Keywords:** exerkine, FNDC5 gene, myokine, exercise

## Abstract

Irisin is an exercise-induced myokine implicated as a fundamental mediator of physical activity benefits. The aim of the present study was to investigate the role of the chronic administration model of irisin on the physiological and molecular status of skeletal muscle. A total of 20 female Sprague Dawley rats (250 ± 40 g) were implanted with an irisin-loaded osmotic pump (5 µg/kg/day) for 42 days; in addition, 3 females received a single subcutaneous injection of irisin (5 µg/kg). On a weekly basis for six weeks, animals were weighed and blood samples were collected. After 42 days, hind muscle biopsies were collected for histology and gene analysis. Serum irisin, clinical biochemistry, and histopathology were quantified and evaluated. Genes encoding for different physiological muscle activities, such as oxidative stress, fatty acid metabolism, muscle hypertrophy, mitochondrial fusion, and aging were assayed. The results showed a significant reduction in body weight percentage and creatine kinase level without affecting the morphological characteristics of skeletal muscle. Significant changes were noted in genes involved in muscle physiological activity, growth, and aging, as well as genes encoding for the antioxidant system, fatty acid oxidation processes, and mitochondrial fusion. In conclusion, exogenous irisin can induce the same physiological and molecular mechanisms that might be induced by exercise.

## 1. Introduction

Quality of life is undoubtedly improved by a fit lifestyle. Regular physical activity can enhance our health and reduce the risk of several disorders, such as type 2 diabetes, cancer, and cardiovascular disease. Recently, skeletal muscles were recognized as an endocrine organ that produces myokines such as irisin (fibronectin type III domain-containing protein 5 (FNDC5)) [1]. A novel myocyte-secreted hormone, irisin, was suggested to mediate some of the useful effects of exercise such as browning of adipocytes, thermogenesis, prevention of weight gain, and metabolic homeostasis [2]. Nevertheless, the correlation between increased risk factors of many diseases and aging where decreased muscle mass and impaired muscle function (sarcopenia) are dominant is still ambiguous [1].

Irisin is a myokine secreted from skeletal muscle in response to exercise both in rats and humans. Researchers originally identified irisin as a myokine that targets white adipocytes to induce browning response and, subsequently, non-shivering thermogenesis, in addition to its role in the control of bone mass [3,4,5]. Recently, it was revealed that circulating irisin increases in a transient manner via an acute intention to exercise and that its concentration correlates with exercise intensity. Nevertheless, irisin is one of the peripheral exerkines and has been reported to reduce anxiety following exercise training [6].

A previous study evaluating the effect of irisin on the cardiac muscle at relaxed conditions found improvement in cardiac health and biomarkers. Therefore, the current study aimed to investigate the impact of chronic irisin administration (using surgically implanted osmotic pumps) on the skeletal muscle gene expression as well as the muscle physiology, body weight, and biomarkers of normal unstressed rats.

## 2. Materials and Methods

### 2.1. Experimental Design

To exclude the action of male anabolic steroids, female Sprague Dawley rats (250 ± 40 g) were utilized in the current investigation and housed at the Laboratory Animal Research Unit of the University of Petra Pharmaceutical Center, Amman, Jordan. Animals were kept under controlled temperatures of 23 ± 1 °C, humidity of 60 ± 5%, and a 12-h light/12-h dark cycle. All experiments were conducted in accordance with the Institutional Guidelines of the University of Petra, which adopts the Federation of European Laboratory Animal Science Association (FELASA) guidelines.

### 2.2. Pump Implantation and Irisin Administration

Animals were acclimated and then separated into two groups (*n* = 10): a sham group receiving a bare surgery and another receiving an ALZET Osmotic Pump 2006 (Cupertino, CA, USA) loaded with recombinant human irisin (FNDC5) (CusaBio, Houston, TX, USA) at a concentration of 0.25 μg/μL. The surgery and implantation occurred at the rats’ dorsal area under anesthesia using isoflurane (Hikma Pharmaceuticals, Amman, Jordan), as described briefly in a previous publication [7].

### 2.3. Sampling

Animals were observed daily for 42 days and weighed. Blood samples were withdrawn weekly from the ocular sinus using withdrawn heparinized capillary tubes. Serum was analyzed at MedCare Labs (Madaba, Jordan). Muscle biopsies from the hind region were collected for histopathology and RNA extraction.

### 2.4. Irisin Quantification and Kinetics

Another three female rats were acclimated and used to determine the kinetics of irisin and quantification in serum. Rats received a bolus subcutaneous injection of irisin at a dose of 5 mg/kg, equivalent to a daily dose released through the pump. Afterwards, animals were subjected to blood sampling at specific time intervals (0–4 h) and serum was separated for quantification using ELISA analysis. Irisin levels were quantified using the Irisin ELISA kit (CusaBio, Houston, TX, USA) in accordance with the manufacturer’s instructions.

### 2.5. Superposition Principle Simulation Modeling

Superposition principle simulation modeling was generated using irisin quantification levels of animals administered irisin subcutaneously compared to levels obtained from animals administered irisin via pump. The WinNonlin noncompartmental analysis program (Version 5.2) was used for the analysis. Representative figures for the results were retrieved from the original published article [7].

### 2.6. Histopathology

Hind muscle tissue samples were collected 42 days after pump implantation and preserved in formalin. Next, samples were processed, embedded, and stained using hematoxylin and eosin (H and E) stains. Slides were then visualized using light microscopy at 40× magnification.

### 2.7. RNA Isolation, cDNA Synthesis, and RTq-PCR

At day 42, hind muscle tissue was collected from the animals and snap frozen (using liquid nitrogen) for further RNA extraction. Homogenized samples were then subjected to isolation of RNA using a Trizol reagent followed by full RNA extraction using Direct-ZolTM RNA MiniPrep (Zymo Research Co., Irvine, CA, USA). Samples were tested for integrity and quantity using fluorometry and spectroscopy measurements. Thereafter, RNA samples were reverse transcribed and run in RTq-PCR to analyse target genes. Gene analysis parameters and selected primer sequences were as described elsewhere [7].

## 3. Results

### 3.1. Body Weights

**Figure 1:**
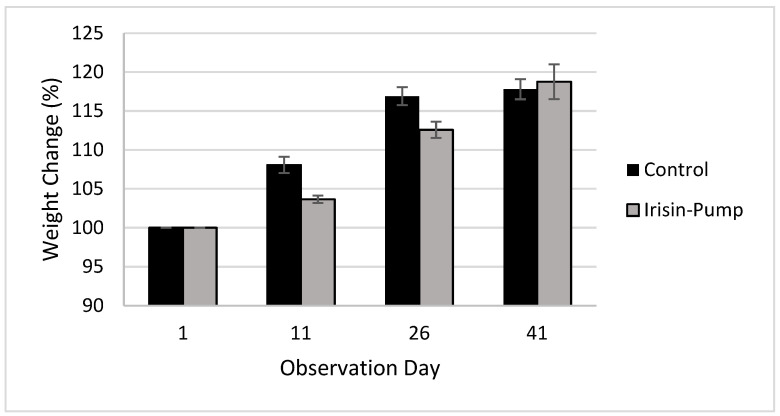
Percent weight change in Sprague Dawley rats after chronic administration of irisin via an osmotic pump.

A decrease in body weights of rats receiving irisin on a chronic basis was noted, as observed in Figure 1. Such change was considered clinically significant in comparison to animals that were not treated with irisin.

### 3.2. Clinical Biochemistry

Although statistically insignificant, a drop in LDH levels was noted in animals receiving irisin (Table 1). On the other hand, a significant decrease in creatine kinase levels was also observed. Serum levels of troponin were unaffected.

### 3.3. Quantification of Serum Irisin

**Figure 2:**
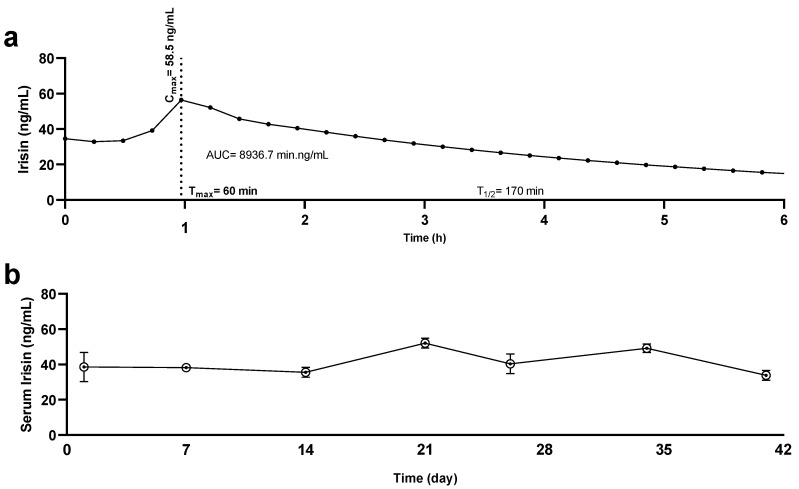
Pharmacokinetic profiles of irisin. Serum sampling and irisin quantification of Sprague Dawley rats administered irisin via (**a**) a subcutaneous route and (**b**) an osmotic pump. Dotted line represents time (T_max_) it takes for irisin to reach the maximum concentration (C_max_).

A pharmacokinetic profile was generated by determining irisin levels in animals that were administered irisin subcutaneously. A peak in irisin levels was detected in serum 1 h after dosing (58.5 ng/mL), as shown in Figure 2. The presented data are briefly described in a previous publication [7]. As shown in Figure 2b, levels of irisin increased after 14 days and returned to normal at day 42; such results are in line with the pump’s design, which is intended to deliver its contents for up to 42 days, as labelled by the manufacturer.

### 3.4. Histopathology

**Figure 3:**
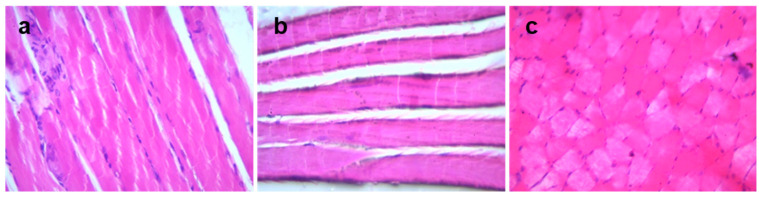
Morphological evaluation of hind muscle of irisin-treated rats. Hind muscle tissue sections of (**a**) control animals and (**b**,**c**) irisin (pump)-treated animals (×40).

The morphology of hind muscle tissue was observed after H and E staining; intact and consistent muscle architecture was observed in the tissue of both control and irisin-receiving animals (Figure 3).

### 3.5. Gene Expression Levels

**Figure 4:**
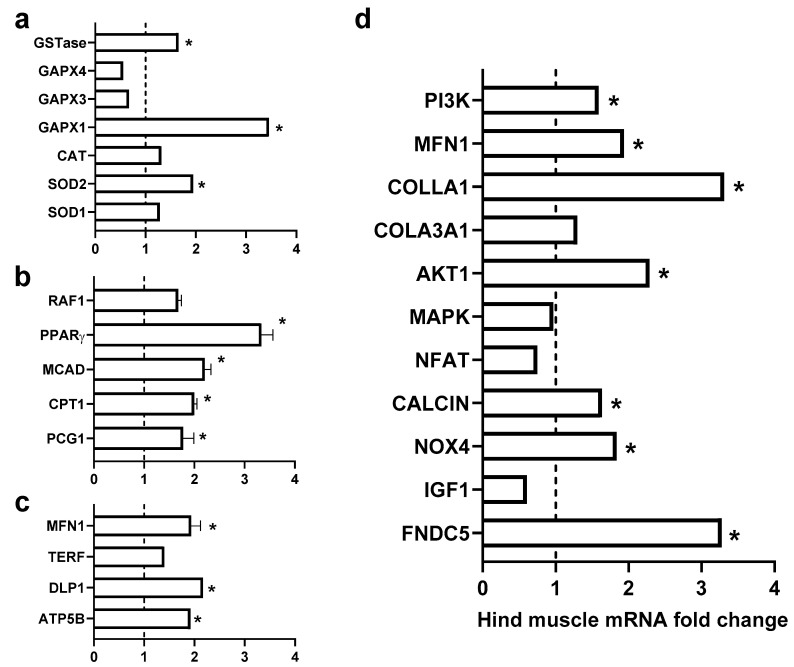
Effects of chronic administration of exogenous irisin (5 mg/kg/day) for 42 days on heart mRNA levels. (**a**) Genes encoding against oxidative stress; (**b**) genes encoding for fatty acid β-oxidation activities; (**c**) mitochondrial fusion; and (**d**) genes encoding for muscle mass and aging. * *p* < 0.05 in comparison to the control group.

The majority of studied target genes were found to be upregulated after treatment with irisin. As observed in Figure 4, an increase in genes encoding for myocyte homeostasis and activity was observed, and others encoding against oxidative stress were elevated. In addition, other genes mediating cell metabolism and mitochondrial activities were also altered.

## 4. Discussion

Exercise has a significant beneficial effect on individuals’ physical function at all ages. Given that skeletal muscle is an abundant resource of irisin, an exercine exerted during exercise, the current study hypothesized that exogenous exposure to irisin would induce the same effect in individuals without engaging in physical activities. The introduction of osmotic pumps in individuals with low levels of physical activity could enhance the quality of their muscles and health. The present study measured the percentage change in body weight and clinical serum biomarkers including myoglobin, creatine kinase, troponin-t, and lactate dehydrogenase. A clinically significant decrease in body weights was observed in animals treated with irisin; however, our histological results showed no difference in myocytes in both groups, suggesting that weight loss did not affect muscle tissue. In addition, the significant decrease in serum creatine kinase suggests an absence of muscle damage. The negative correlation between serum irisin levels and creatine phosphokinases has been reported in the literature and proposed as an accurate biomarker, along with irisin, in cases of myocardial pathologies [8]. Nevertheless, correlating whether irisin directly induces a decrease in CK levels or if an increase in irisin is spontaneously acquainted with a decrease in CK levels is still elusive.

Our study also measured the expression of selected genes in response to interventional exogenous irisin. Those selected genes involved physiological activity in muscles, such as oxidative stress, fatty acid metabolism, muscle hypertrophy, mitochondrial fusion, and aging.

### 4.1. Role of Exogenous Irisin in Oxidative Stress

The expression of genes (including SOD1, SOD2, Catalase, GPX1, GSTase, and NOX4 (Figure 4a)) encoding against oxidative stress was activated in muscles of animals treated with irisin. It is well-known that physical activity enhances the antioxidant enzyme activities of super-oxide dismutase (SOD) and glutathione peroxidase (GPx) in skeletal muscle [9,10,11]. Nevertheless, the change in expression of antioxidant encoding genes could be attributed to a potential antioxidant and beneficial effect of irisin on cell physiology. Knowledge regarding the antioxidant effects of irisin is very recent and new. Current findings are in line with a few studies that were suggestive of its antioxidant properties and beneficial outcomes in the redox system, specifically on the NRF2/HMGB1 axis, which is a key modulator of a cascade of antioxidant molecules [12,13,14]. Therefore, it could be concluded that interventional exogenous irisin can induce the same effect as exercise on skeletal muscles and increase the gene expression of several antioxidant proteins, including Cu/Zn superoxide dismutase (SOD1), manganese-dependent superoxide dismutase (SOD2), catalase, glutathione peroxidase 1 (GPX1), glutathione S-transferases (GSTs), and NADPH oxidase 4 (NOX4). Notably, these antioxidative enzymes play essential roles in protecting cells from ROS damage, with a remarkable decrease in damage markers after exercise [15]. Moreover, a very recent study showed that muscle catalase could prevent neuromuscular junction disruption and its related muscle atrophy in SOD1 KO mice [16], suggesting the importance of this enzyme in muscle pathophysiology and supporting the importance of irisin in enhancing the gene expression of catalase in muscles, thereby protecting the muscles. The current investigation reported a significant increase in GSTase, a well-known enzyme responsible for GSH conjugation, which suggests its important role in muscle tissue detoxification [17,18]. The exogenous irisin also increased the NOX4 gene level in skeletal muscle, which is an extremely important gene due to its role in facilitating ROS-mediated adaptive responses in the skeletal muscle that boost muscle function, preserve redox balance, and prohibit the development of insulin resistance [19].

### 4.2. Role of Exogenous Irisin in Fatty Acid β-Oxidation FAO

It is well-known that 80% of energy demand is provided by the oxidation of fatty acids during fasting. The major source of energy in both skeletal muscle and myocardium utilizes long chain fatty acids through a beta-oxidation system [20]. Our results showed a significant increase in the level of gene expression for important genes that regulate the fatty acid oxidation process, specifically PPAR- γ, CPT1, and MCAD (Figure 4b). Peroxisome proliferator-activated receptor type γ (PPAR-γ) is one of the nuclear hormone receptor super families that play a vital role in glucose and fatty acid metabolism [21]. On the other hand, carnitine palmitoyltransferase 1 (CPT1) and medium-chain acyl-CoA dehydrogenase (MCAD) are key regulatory enzymes in mitochondrial fatty acids oxidation, which is the main muscle energy source during endurance exercise [21]. Our results are in accordance with findings from a previous study [21] that swimming exercise can activate PPAR-γ expression in the liver tissue and simultaneously increase CPT-1 and MCAD levels, and thereby relieve liver lipid disorders in insulin-resistant mice.

### 4.3. Role of Exogenous Irisin in Mitochondrial Fusion

Physical exercise significantly promotes useful skeletal muscle adaptations, ranging from increased endurance due to mitochondrial biogenesis, fusion, and angiogenesis to increased strength due to hypertrophy [22]. Mitochondrial fusion is a very important physiological activity that boosts mitochondrial homogenization by content mixing and thereby maintaining mitochondrial function [22]. The importance of mitochondrial fusion is reported by several studies, as mutations in fusion genes lead to neurodegenerative disease in humans and severe defects in many mice cell types [23,24]. Our results showed a significant increase in the level of gene expression of DPL1 and Mfn, which are the main regulatory genes for mitochondrial fusion, suggesting that the interventional irisin may have protected mitochondrial function by promoting mitochondrial fusion (Figure 4c).

### 4.4. Role of Exogenous Irisin in Muscle Mass and Aging

Muscle pathological and physiological adaptations are regulated in response to changes in physical activities and pathological conditions. Muscle mass is controlled by maintaining a balance between anabolic and catabolic processes [25]. Muscle growth during hypertrophy is regulated at both the translational and transcriptional levels through the prompting of protein synthesis and activation of ribosomal RNAs and muscle-specific genes [26]. The present study showed a significant increase in the level of gene expression of many genes that induce muscle growth, including AKT1 and PI3K, which were reported to increase protein synthesis in muscle cells [25,26,27]. Our results suggest that irisin is a potential candidate for reducing muscle loss in patients suffering from atrophy and thus incapable of physical exercise. However, more studies will be needed to substantiate this suggestion. On the other hand, a significant increase in the expression of genes that reduce the influence of muscle’s aging process, including COL3A1, COL1, and TERF, were hereby reported. The mechanisms of aging are complex, but there is a belief that various genes encode proteins that can enhance quality of life by either “turning on” or “turning off” genes acting as antioxidants, regulating metabolisms by turning on genes that prompt stress resistance, collagens, and metabolism. It could also turn on genes that enhance stress response, fertility, and cell growth [27].

Interventional irisin induced the expression of genes that fight stress products such as SOD1, SOD2, catalase, GPX1, GSTase, and NOX4. Our results also showed a significant increase in metabolic regulatory genes, namely PGC1, PPAR- γ, CPT1, and MCAD (Figure 4d). Moreover, the present study reports increased expression levels of genes responsible for mitochondrial fusion and hypertrophy adaptations, suggesting a decrease in muscle loss. To our knowledge, this is the first study that investigated the novel question of the potential role of interventional irisin as a chronic model in skeletal muscle physiology. This research might be considered preliminary and further experimental studies are needed, including muscle mass and grip strength assessments, and proteomic and metabolomics analyses. We hope that this study will motivate further studies in this area; its results have potentially far-reaching implications for novel approaches to pharmacologic modulation in skeletal muscle pathogenesis in response to cancer, degenerative disease, and aging.

## 5. Conclusions

Interventional irisin administrated in a chronic model showed a beneficial effect on molecular and physiological levels of rat muscle. In addition to reducing muscle damage, exogenous irisin reduced the weight of rats without affecting muscle morphology. Molecular evaluation results showed the prompted expression of many genes involved in different physiological muscle activities, such as oxidative stress, fatty acid metabolism, muscle hypertrophy, mitochondrial fusion, and aging. The impact of irisin in healthy animals is evident; however, its effect on exercised and physically stressed animals is also of great importance and is worthy of further investigation. Nevertheless, further studies and trials are required to integrate the use of osmotic pumps and patches loaded with irisin for different medical outcomes.

## Figures and Tables

**Table 1:** Serum clinical biochemistry.

Clinical Biochemistry Parameters	Control	Irisin Pump
Myoglobin (nmol/L)	110.4 ± 30.4	129.6 ± 41.8
Creatine kinases (U/L)	364 ± 25.2	256.8 ± 39.5 *
Troponin-T (ng/mL)	1655.5 ± 568.3	1713.5 ± 482.6
Lactate dehydrogenase (LDH) (U/L)	1335.4 ± 223.3	808.2 ± 156.7

Mean ± SEM; * *p* < 0.05; analyzed using Mann–Whitney non-parametric analysis.

## Data Availability

Data are contained within the article.
